# Comparison of Heavy-Duty Scuffing Behavior between Chromium-Based Ceramic Composite and Nickel-Chromium-Molybdenum-Coated Ring Sliding against Cast Iron Liner under Starvation

**DOI:** 10.3390/ma10101176

**Published:** 2017-10-14

**Authors:** Yan Shen, Baihong Yu, Yutao Lv, Bin Li

**Affiliations:** Marine Engineering College, Dalian Maritime University, Dalian 116026, China; 18041103373@163.com (B.Y.); a0330lt@163.com (Y.L.); libin1992dlmu@163.com (B.L.)

**Keywords:** heavy-duty scuffing, piston ring coating, cast iron cylinder liner, starvation

## Abstract

A running-in and starved lubrication experiment is designed to investigate the heavy-duty scuffing behavior of piston ring coatings against cast iron (Fe) cylinder liner using the piston ring reciprocating liner test rig. The scuffing resistance of the piston ring with the chromium-based ceramic composite coating (CKS), and that with the thermally sprayed nickel-chromium-molybdenum coating (NCM) is compared at different nominal pressures (40~100 MPa) and temperatures (180~250 °C). With the failure time as a criterion, the rank order is as follows: NCM/Fe > CKS/Fe. Before the scoring occurs at the interface of the piston ring and cylinder liner (PRCL), the cast iron liner enters into a “polish wear” stage, and iron-based adhesive materials begin to form on the piston ring surface. With the macroscopic adhesion formation, the plastic shearing cycle causes surface damages mainly due to abrasive effects for the CKS/Fe pairs and adhesive effects for the NCM/Fe pairs.

## 1. Introduction

As one of the main frictional pairs in the engine, the piston ring and cylinder liner have direct impacts on the engine’s mechanical efficiency and service life. With the development of the internal combustion engine in high power density, scuffing problems under severe working conditions have received more attention [1,2].

Scuffing is often recognized as an abrupt rise in the coefficient of friction accompanying by a sudden increase in noise and vibration. Many efforts have been made to investigate the scuffing phenomenon from different perspectives. Blok gave a criterion to judge the initiation of scuffing with the critical temperature, which was the sum of the bulk temperature and the flash temperature generated in the contact area [3]. Czichos extended the critical-temperature theory with the critical interfacial energy to judge the failure of the lubricated concentrated contacts [4]. Rabinowicz suggested that the surface roughening and size of wear particles formed during sliding caused galling seizure occurrence [5]. Ludema proposed that wear debris agglomeration led to scuffing [6]. Enthoven and Spikes suggested that the onset of scuffing was always immediately preceded by the buildup of fine wear debris in the contact inlet [7]. Saeidi proposed that scuffing initiated when the tribo-film iron oxide reduced to iron and metal-metal contact and, thus, adhesion took place [8]. Yagi observed the phase transformation of steel in the scuffing process under dry conditions, and the flattening of the whole contact area and dramatic expansion with changing the conformity seemed to play important roles in scuffing [9,10]. Yagi also investigated the overall wear process with a combination of two-dimensional detector synchrotron X-ray diffraction (XRD), a near-infrared charge-coupled device (CCD) array, and a visible CCD array, and identified the friction stages as first micro-scuffing, normal wear, second micro-scuffing, and macro-scuffing, respectively [11]. Wojciechowski proposed that the “oleophilic” and “oleophobic” properties of metallic surfaces as autonomous invariants determined the activation of the catastrophic wear process under boundary lubricated conditions [12]. Kamps revealed that mild scuffing produced a smooth surface with small cracks. The transition to severe scuffing occurred when crack networks facilitated the adhesive transfer of cast iron material to the counter-surface [13]. Ajayi developed a predictive analytical model for scuffing as the basic mechanism of failure based on the adiabatic shear instability [14]. Despite the extensive efforts to the scuffing problem, the complicated scuffing phenomenon is still not well understood. As the tribological behavior has a systematic dependence and many involved factors, the problem of scuffing in frictional pairs needs more case-by-case analyses, especially in the tribological system of the piston ring and cylinder liner.

Boron phosphorus alloy cast iron with a high performance-price ratio, excellent processing properties, as well as excellent tribological properties has been widely applied in the manufacturing of the engine cylinder liners. A coating on the piston ring may offer advantages such as friction reduction, wear resistance, and better scuffing performance. Öner examined the structural changes on the CrN-coated surface due to thermal and mechanical shocks [15]. Jisheng investigated the sliding wear behavior of NCM-coated steel under lubricated conditions [16]. The test results of Zeng showed that the wear resistance of Cr coatings electrodeposited with Al_2_O_3_ particles was improved remarkably [17]. Lin prepared TiSiCN nanocomposite coatings which showed a 28% and 40% lower ring weight loss for the coated top and second rings, respectively, as compared to the uncoated baseline [18]. Wan discussed the scuffing mechanism of engineered rings by the observation of the damaged characteristics and the chemistry of the rubbing parts with and without a graphite-like carbon surface [19]. With emergence of piston rings with various surface coatings, the scuffing behavior should be further evaluated in poorly lubricated sliding conditions in contact with cast-iron cylinder liners. The anti-scuffing performance should meet the increasing demand of diesel engines under harsh conditions.

The purpose of this paper is to conduct and analyze a series of experiments to compare the heavy-duty scuffing behavior of a grey cast iron liner with typical NCM- and CKS-coated piston rings. It mainly presents the scuffing behavior and failure mechanism of the mating pairs under starved lubrication conditions. This provides a reference for the anti-scuffing design of piston ring coatings.

## 2. Experimental Details

### 2.1. Test Rig Description

**Figure 1:**
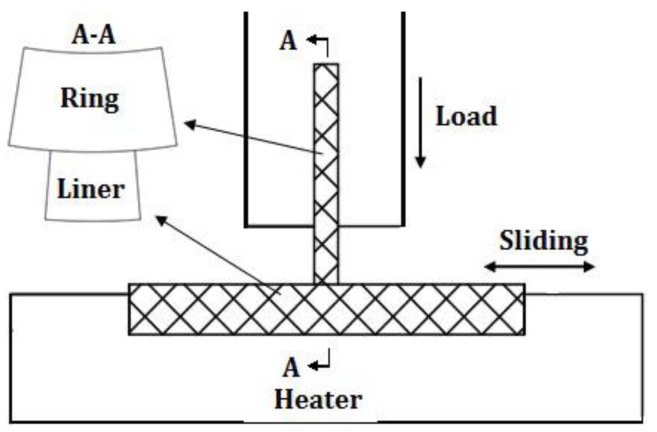
Piston ring reciprocating cylinder liner test rig.

The piston ring reciprocating liner test rig, as illustrated in Figure 1, is designed and constructed for the piston ring and cylinder liner (PRCL). The test rig can accommodate a wide range of nominal pressures (5~380 MPa), speeds (5~500 r/min), and temperatures (25~300 °C) at the interface of the moving specimen (cylinder liner sample) and fixed specimen (piston ring sample). Lubricating oil is applied to the interface through a peristaltic pump. A piezoelectric force transducer and charge amplifier are chosen to measure the horizontal friction force at the PRCL interface.

### 2.2. Experimental Materials

Liner samples utilize boron-phosphorus alloy cast iron + honing. Its roughness Ra (arithmetical mean deviation) is 0.72 μm and its hardness is measured to be an average of 238 HV_0.1_. Its inner diameter is 110 mm, and its thickness is 10 mm. It was cut into 40 equal portions along the circumference. The piston ring has an inner diameter of 70 mm, an outer diameter of 110 mm, and a thickness of 3 mm. It was cut into 20 equal portions along the circumference. 

**Figure 2:**
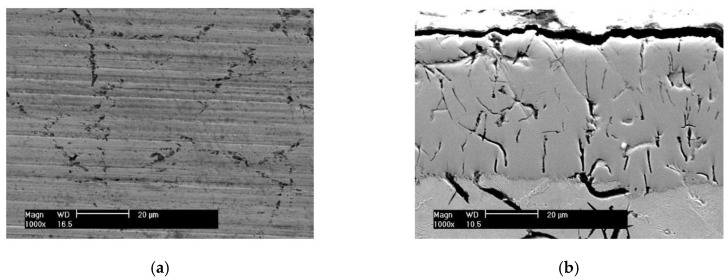
Surface (**a**) and cross-section (**b**) morphology of the piston ring with the CKS coating.

Figure 2 is the surface morphology and cross-section microstructure of the piston ring with the CKS coating. Its roughness Ra is 0.24 μm and its hardness is 705 HV_0.1_. The scattered distribution of non-connected micro-cracks does not extend through the entire chromium coating (about 60 μm in thickness). The fine aluminum oxide particles exist in the micro-cracks. 

**Figure 3:**
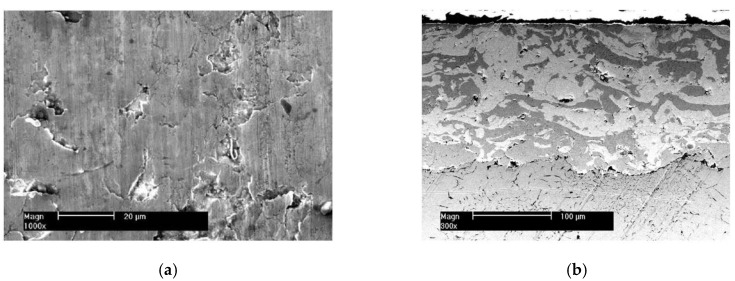
Surface (**a**) and cross-section (**b**) morphology of the piston ring with the NCM coating.

Figure 3 is the surface morphology and cross-section microstructure of the piston ring with the NCM coating. Its roughness Ra is 0.35 μm and its hardness is 535 HV_0.1_. The surface coating is a little rougher with the distribution pits of different size. The coating thickness is about 150 μm. It consists of pure Mo (light grey color) and NiCr alloy (dark grey color). NiCr alloys have self-lubricating properties at high temperatures. The basic material of the above two piston rings is gray cast iron.

To guarantee experimental repeatability, the piston ring has no gap clearance to avoid the effects of the original shape. Mineral-based, fully-formulated engine oil (15W-40), which contains zinc dialkyl dithiophosphates (ZDDP) additives, serve as the lubricating oil in the experiments. Table 1 shows the main element contents of the lubricating oil by the measurement of the Prodigy XP ICP spectroanalysis instrument (Teledyne Leeman Labs, Hudson, NH, USA).

###  2.3. Experimental Procedure

To ensure the accuracy and repeatability of the experiment, the following procedure is used. First, the samples of piston ring and cylinder liner are cleaned with acetone. Second, after the liner sample is fixed, the ring sample is slowly lowered until it comes into contact with the liner sample. The interface between the samples must contact evenly along their arc directions.

The experiment was divided into three stages: the running-in stage with the light load (RLL), the running-in stage with the heavy load (RHL), and the oil starvation stage (OS). The running-in stage was supplied with adequate lubricating oil at the speed of 0.1 mL/min. The oil was evenly distributed along the entire 30-mm stroke. The RLL and RHL stages were provided to eliminate machining defects, such as large burrs, and provide a stable contact state before starvation. At the end of the RHL period, the lubricating oil supply was stopped. Then, starved lubrication of the OS stage occurred at the PRCL interface. The experiment essentially simulates the starvation at the top dead center in a diesel engine. 

Table 2 contains the nominal pressure, temperature, and speed conditions at the interface of the piston ring and cylinder liner. This nominal pressure can be thought of as the sum of the ring elastic pressure and gas pressure acting on the back side of the ring. In the RLL stage, the nominal pressure and temperature were set at 10 MPa and 120 °C, respectively. In the RHL stage, four kinds of nominal pressure (40, 60, 80, and 100 MPa) and temperature (180, 200, 220, and 250 °C) were considered to conduct a comprehensive test. The nominal pressure and temperature conditions of the OS stage were the same as those of the RHL stage. The rotational speed was set at 200 r/min in every test. Three tests were performed to check the results reproducibility for every test condition. 

## 3. Results and Discussion

### 3.1. Friction Force Variation of the CKS Ring and NCM Ring

**Figure 4:**
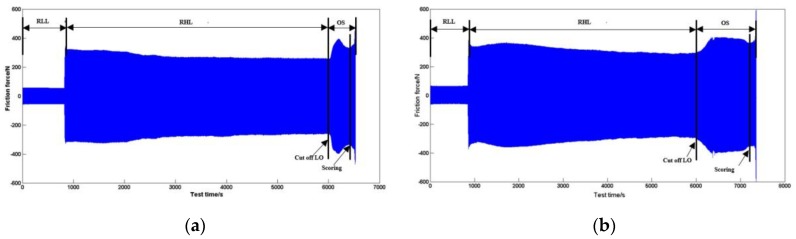
Friction force variation in the starvation experiment (60 MPa, 180 °C): (**a**) CKS ring; (**b**) NCM ring.

Figure 4 shows the friction force variation of the CKS ring and NCM ring in the starvation experiment. Its nominal pressure and temperature in the RHL and OS stage are 60 MPa and 180 °C. The friction force is gradually steady in the latter RHL stage. This trend indicates that the tribological system enters into a stable wear condition. As the lubricating oil is cut off, it enters into the OS stage, and thus the balance of the tribological system is broken. As the residual lubricating oil is consumed continuously at the heavy load, the friction force begins to increase rapidly, then the friction force presents a tendency to decrease gradually and increase sharply. This sharp upward trend of the friction force is always accompanied by harsh noise, and the friction interface results in an obvious scoring on the cast iron cylinder liner. Although the friction force values between the CKS ring and NCM ring are not much difference at the scoring moment, the anti-scuffing time duration in the OS stage is obviously different at the same experimental condition. The method of the friction condition transition by the phase space trajectories is used to acquire the anti-scuffing time duration from the lubricating oil (LO) cutting off to the scoring moment [20]. This time duration can be used to evaluate the anti-scuffing performance of the PRCL for the NCM/Fe and CKS/Fe frictional pairs.

### 3.2. Effect of Nominal Pressure and Temperature on the Anti-Scuffing Time Duration

For the cast iron cylinder liner sliding against the CKS and NCM ring coatings, the scuffing resistance test results on the anti-scuffing time duration as a function of nominal pressure and temperature are shown in Table 3 and Table 4. With the increasing nominal pressure and temperature in the OS stage, the anti-scuffing time duration declines rapidly and then slows down. Through the comparison of the scuffing resistance properties for the CKS/Fe and NCM/Fe pairs at 180 °C, the time duration of the CKS/Fe pairs is less than the NCM/Fe pairs when the nominal pressure is 40 MPa and 60 MPa. The time duration difference becomes smaller at the temperature and nominal pressure exceeding 220 °C and 80 MPa, respectively. Starting from this test condition, the anti-scuffing performance begins to maintain a weak level for the two ring coatings. Through the comparison of a series of the scuffing tests, the scuffing resistance properties of the mating pairs are NCM/Fe > CKS/Fe.

### 3.3. Scuffed Surface Analysis and Discussion

**Figure 5:**
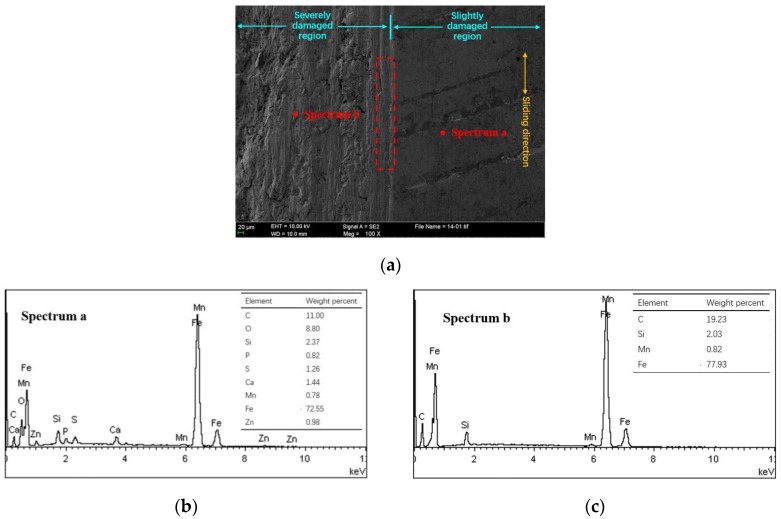
Scuffed cylinder liner surface and elemental content of NCM/Fe. (**a**) Scanning electron microscope (SEM) image; (**b**) EDS spectrum a; (**c**) EDS spectrum b.

Figure 5a presents the scuffed cylinder liner surface of NCM/Fe at 60 MPa and 180 °C. Based on the observation of the slightly damaged region of the scuffed liner, only deeper honing textures still exist on the surface topography, and the platform topography between the honing textures has basically entered into the polishing stage. The energy dispersive spectrometer (EDS) spectra on the platform shows the deposition of S, P, Zn elements originating from the ZDDP extreme additives, as shown in Figure 5b. The area bordering the slightly damaged region shows the abrasive grooves along the sliding direction (red dashed box). A little further from the border area, the bare metal presents without any protective film from the EDS spectra in the severely damaged region as shown in Figure 5c. In addition, compared with the EDS spectra of the severely damaged region, the slightly damaged region contained O elements which may be the oxide film formed to resist the scuffing [8,19]. 

**Figure 6:**
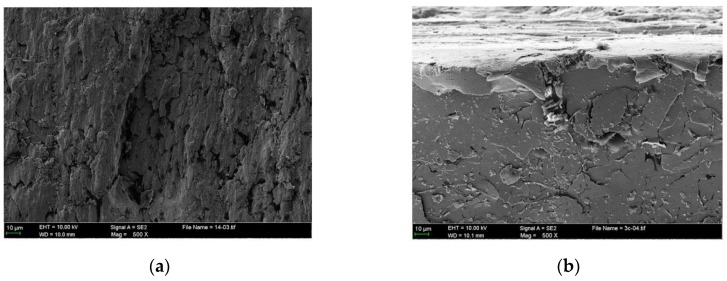
Enlarged worn surface (**a**) and cross section morphology (**b**) of severely damaged region from the scuffed liner of NCM/Fe.

Figure 6a presents the enlarged severely damaged region. It can be seen that the adhesive materials are pulled out, forming several micro-holes that vary in size. Meanwhile, a series of grooves caused by plastic shearing also results in the material removal. As a result of the plastic flow of the cast iron material, the plowing process causes material displaced from a groove to the sides with the ridge formation. The ridges are also flattened by the adhesive asperities. These processes finally form the wear particles due to the reciprocated shearing motion, as shown in Figure 6b. From the cross-section microstructure of the scuffed liner subsurface, it can be seen that severe plastic deformation occurs in the subsurface, while cracks typically occur in the surface and subsurface.

**Figure 7:**
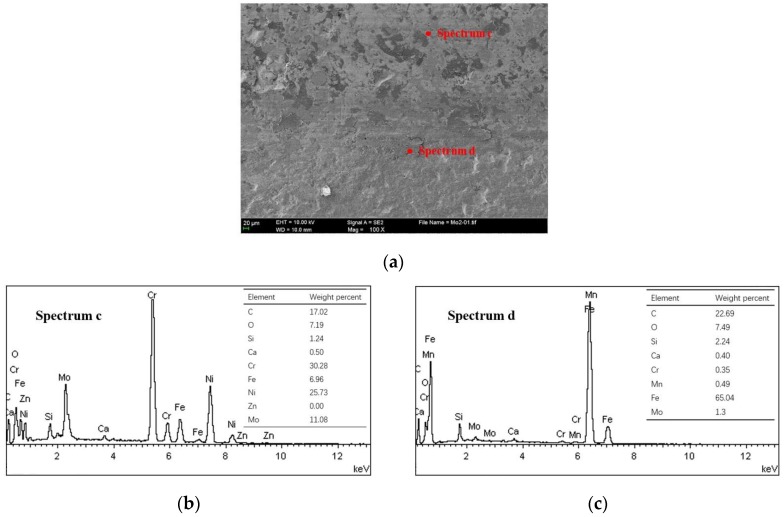
Scuffed piston ring surface and elemental content of NCM/Fe. (**a**) SEM image; (**b**) EDS spectrum c; (**c**) EDS spectrum d.

**Figure 8:**
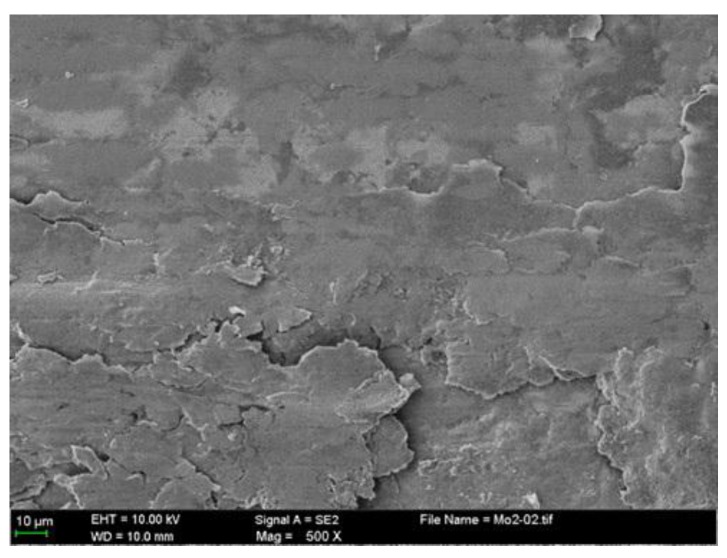
Enlarged worn surface from the NCM ring.

Some transferred cast iron patches result in adhesion on the NCM ring surfaces, as shown in Figure 7a,b. The ring surface distributes the scattered patches with different sizes. The severely damaged areas of the NCM ring are coated with iron-based material, as shown in Figure 7c. This make an obvious boundary between different damaged areas, just like the cast iron surface. The larger patches seem to be the loose particle agglomerates flattened on the ring surface, as shown in Figure 8.

**Figure 9:**
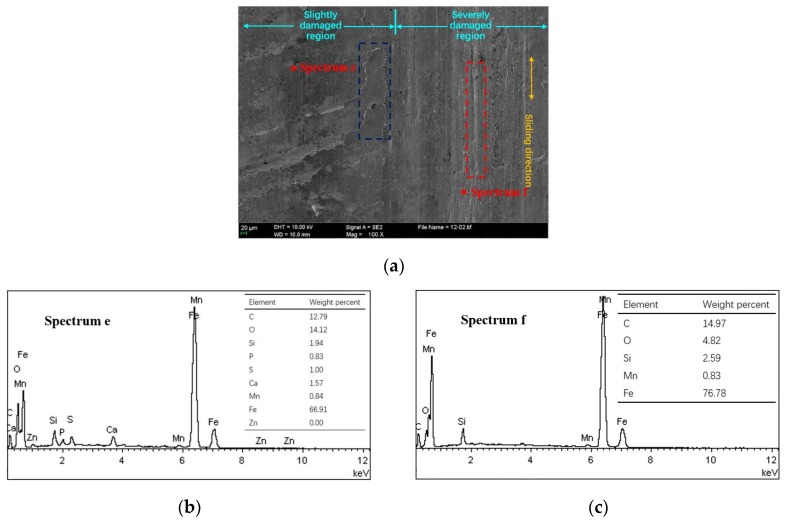
Scuffed cylinder liner surface and elemental content of CKS/Fe. (**a**) SEM image; (**b**) EDS spectrum e; (**c**) EDS spectrum f.

Figure 9a presents the scuffed cylinder liner surface of CKS/Fe at 60 MPa and 180 °C. The honing textures on the slightly damaged region experienced a plastic flow under the local high stresses and frictional heat [11,21]. More flake debris appeared on the surface along the sliding direction (blue dashed box). Through the EDS spectrum, it can be seen that the slightly damaged region contains small amounts of extreme pressure additive elements, S, P, and Zn, as shown in Figure 9b, but none of the additive elements exist in the severely damaged region, as shown in Figure 9c. The bare metal on the severely damaged surface shows the plastic deformation of the furrows (red dashed box).

**Figure 10:**
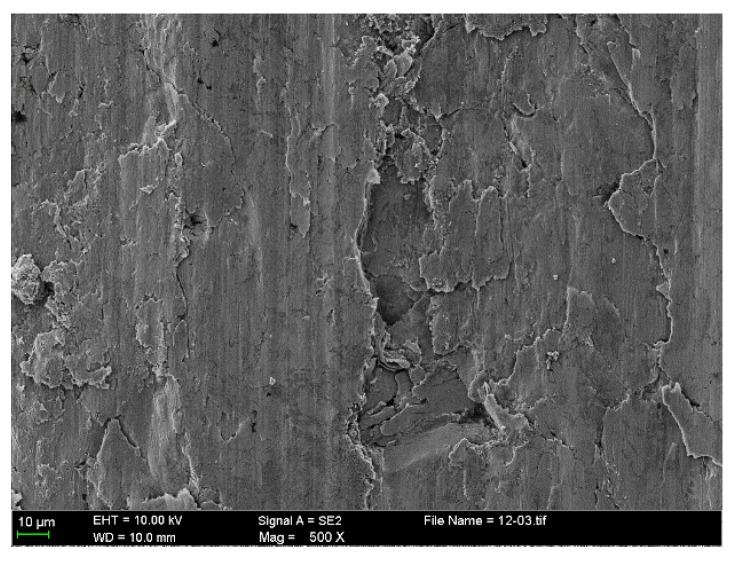
Enlarged worn surface of severely damaged region from the scuffed liner of CKS/Fe.

**Figure 11:**
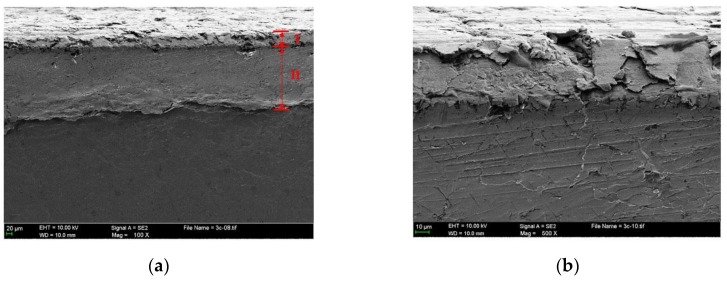
Cross section overview (**a**) and magnified (**b**) morphology of severely damaged region from the scuffed liner of CKS/Fe.

Figure 10 presents the enlarged severely damaged region. It shows that the macro adhesion tends to cause subsurface plastic deformation and may contribute to the nucleation of surface and subsurface cracks. Further shearing cycles cause these cracks and pre-existing voids and cracks to propagate, even expanding to the subsurface, as is shown in Figure 11. The subsurface can be divided into two deformed layers with different thickness, as shown in Figure 11a. In the severely deformed layer (I), the flake debris cracks or falls off in the defect or stress concentration area. Some wear fragments detach from the surface with the propagation of a shear crack, as shown in Figure 11b. The slightly deformed layer (II) mainly presents the deformed traces caused by the reciprocating motion. 

**Figure 12:**
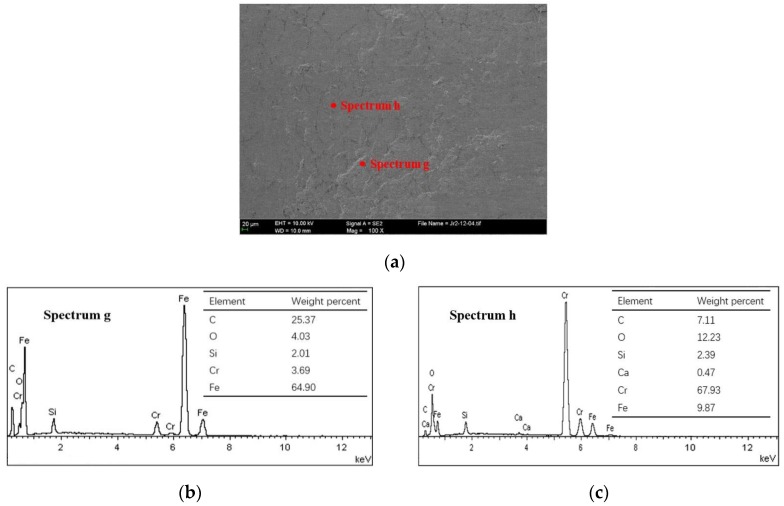
Scuffed piston ring surface and elemental content of CKS/Fe. (**a**) SEM image; (**b**) EDS spectrum g; (**c**) EDS spectrum h.

**Figure 13:**
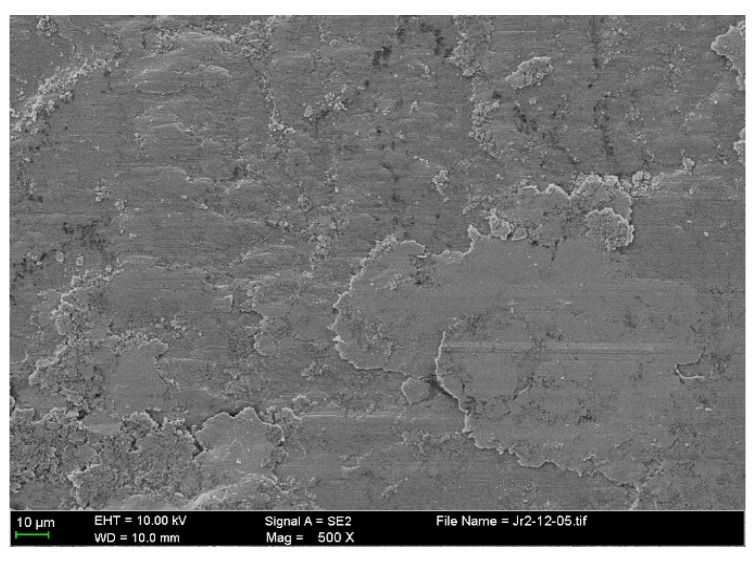
Enlarged worn surface from the CKS ring.

The worn patches are transferred to the mating surface, as shown in Figure 12. Cast iron debris of varying size scatters over the piston ring surface. From the enlarged debris accumulation area in Figure 13, the transferred large cast iron patches seem to be fractured into many fine patches and stick to the piston ring surface by the heavy crush. This may be the main cause of why the anti-scuffing duration of the CKS ring is less than that of the NCM ring. Many fine abrasive particles on the CKS ring surface prevent the uniform distribution of the lubricating medium to the asperities contact area [22]. A stress concentration zone will be formed, thus resulting in more scuffing initiation spots.

Based on the analysis of the scuffed PRCL, the scuffing sequence of the damage to the final failure seems to involve the following events for the two piston ring coatings:

(a) The surface honing textures of the cast iron cylinder liner gradually becomes shallow and its friction condition is in the “polishing wear” stage.
(b) The formation of microscopic and macroscopic adhesions causes cast iron plastic shearing, resulting in different damage modes of the surface materials transfer. Meanwhile, the subsurface accompanied with the deformation contributes to the formation of abrasive particles.
(c) Most transferred materials adhere to the piston ring surface with different particle aggregation patterns.

## 4. Conclusions

Heavy-duty scuffing behavior of the PRCL interface is obtained using the piston ring reciprocating liner test rig. The interface contacts are starved of lubrication after the running-in period. The effects of nominal pressures and temperatures on the scuffing resistance of the piston ring coatings are evaluated. The experimental results can be summarized as follows:

(1) When mated with a cast iron cylinder liner, the NCM coating has better scuffing resistance properties than the CKS coating with the failure time criterion.
(2) At the heavy-duty sliding conditions, when the nominal pressure and temperature exceed more than 60 MPa and 220 °C, respectively, the anti-scuffing performance exhibits an abrupt downward trend. Exceeding these harsh conditions, the anti-scuffing performances are maintained at a weak level.
(3) Before the scoring occurs at the PRCL interface, the cast iron liner enters into a “polish wear” stage, and iron-based adhesive materials begin to form on the piston ring surface.
(4) The plastic shearing cycle causes surface damages mainly due to adhesive effects for the NCM/Fe pairs, and abrasive effects for the CKS/Fe pairs.

## Figures and Tables

**Table 1:** Main element contents of the lubricating oil.

Elements	Fe	Cu	Al	Pb	Ni	Si	Ca	Mg	P	Zn
Content (ppm)	24	13	5	3	7	19	7200	21	757	406

**Table 2:** Experimental conditions.

Experimental Stage	Nominal Pressure (MPa)	Speed (r/min)	Temperature (°C)	Time (min)
RLL	10	200	120	~15
RHL	40, 60, 80, 100	200	180, 200, 220, 250	~85
OS	40, 60, 80, 100	200	180, 200, 220, 250	To scuffing

**Table 3:** Anti-scuffing time duration of the CKS/Fe pair *.

Temperature (°C)	Nominal Pressure (MPa)			
	40	60	80	100
180	36.0 ± 1.0	18.7 ± 2.7	6.0 ± 0.0	5.7 ± 0.7
200	29.7 ± 1.7	13.3 ± 2.3	5.3 ± 0.7	3.3 ± 0.7
220	9.3 ± 1.7	5.0 ± 0.0	4.7 ± 0.7	2.7 ± 0.7
250	5.3 ± 0.7	4.0 ± 0.0	3.3 ± 0.7	2.3 ± 0.7

* The unit of time duration is min.

**Table 4:** Anti-scuffing time duration of the NCM/Fe pair *.

Temperature (°C)	Nominal Pressure (MPa)			
	40	60	80	100
180	79.3 ± 3.7	22.3 ± 1.7	11.0 ± 1.0	5.7 ± 0.7
200	32.7 ± 2.7	13.7 ± 1.3	6.3 ± 0.7	5.0 ± 0.0
220	10.7 ± 1.7	8.7 ± 0.7	5.3 ± 0.7	3.7 ± 0.7
250	7.3 ± 0.7	8.3 ± 0.7	5.0 ± 0.0	3.3 ± 0.7

* The unit of time duration is min.
